# Hypoxia-preconditioned gingiva-derived mesenchymal stem cell-conditioned medium accelerates burn wound healing

**DOI:** 10.1038/s41598-026-54462-8

**Published:** 2026-05-25

**Authors:** Xingyu Zhao, Xuanjia Li, Chengzhe Yang, Bing Wang

**Affiliations:** 1 Department of Plastic, Aesthetic and Burn Surgery, Qilu Hospital of Shandong University, Jinan, Shandong China; 2 Cheeloo College of Medicine, Shandong University, Jinan, Shandong China; 3 Research Center for Basic Medical Sciences, Qilu Hospital of Shandong University, Jinan, Shandong China; 4 Department of Oral and Maxillofacial Surgery, Qilu Hospital of Shandong University, Jinan, Shandong China

**Keywords:** Burn wound healing, Gingival mesenchymal stem cells, Hypoxic preconditioning, PI3K/AKT signalling, Conditioned medium, Immunomodulation, Cell biology, Stem cells

## Abstract

Burn injuries remain a major clinical challenge due to the complex wound microenvironment and limited donor skin availability. Gingival mesenchymal stem cells (GMSCs), which are easily accessible and possess strong immunomodulatory and regenerative capacities, represent a promising therapeutic resource. Increasing attention has focused on the use of GMSC-derived conditioned medium (CM) as a cell-free alternative, particularly when its efficacy is enhanced through hypoxic preconditioning. In this study, human-derived GMSC-conditioned medium was used to examine the effects of hypoxia-preconditioned GMSC-CM (HP-GMSC-CM) on burn wound healing in vitro and in vivo. In HaCaT keratinocyte cultures, HP-GMSC-CM significantly promoted scratch wound closure and enhanced cell migration in Transwell assays, accompanied by upregulated expression of LAMC2 and COL4A1. In M1-polarized macrophages, HP-GMSC-CM markedly suppressed secretion of the pro-inflammatory cytokines TNF-α and IL-1β. In a murine burn model, treatment with HP-GMSC-CM accelerated wound closure and increased the expression of epithelial marker Pan-CK and extracellular matrix protein TNC. Mechanistic inhibition with LY294002 suggested that these therapeutic effects are mediated, at least in part, through **PI3K/AKT-associated signaling**. Collectively, these findings suggest that **hypoxia-preconditioned** GMSC-derived CM may serve as an effective cell-free therapeutic strategy for promoting burn wound repair and support its potential translational applicability despite the use of a xenogeneic experimental model.

**Supplementary Information:**

The online version contains supplementary material available at 10.1038/s41598-026-54462-8.

## Introduction

Burn injuries represent a major global health burden, with approximately 11 million people receiving medical treatment for burns annually^1^. Prognosis is closely related to burn severity: minor burns often heal spontaneously, whereas severe burns—typically defined as those involving > 30% of total body surface area (TBSA) or full-thickness burns affecting > 10% TBSA^2^—can be life-threatening. Over recent decades, advances in burn and critical-care medicine have shifted clinical goals from improving survival to enhancing functional recovery. Alongside fluid resuscitation, infection control, and systemic support, effective wound repair remains important to achieving long-term quality of life in burn survivors. Current research focuses on accelerating wound closure, improving scar quality, and preserving tissue function^3^. The wound-healing process after burn injury progresses through overlapping phases—inflammation, cell recruitment, matrix deposition, epithelialisation, and remodelling^4^. Management of the early inflammatory phase is crucial, as dysregulated inflammation in severe burns can cause excessive scarring and functional impairment, with potential aesthetic and oncogenic consequences^5^. For deep partial- and full-thickness burns, the standard treatment remains eschar excision followed by autologous split-thickness skin grafting, which significantly reduces mortality, shortens hospitalisation, and enhances healing^6^. However, extensive burns continue to pose challenges due to limited donor sites and the complex wound microenvironment, often leading to delayed healing, infection, pain, and hypertrophic scarring^4^. Although autologous epidermal cell transplantation partially alleviates donor-skin shortages, its efficacy is often limited by poor graft survival and infection risk^7^. Stem-cell-based therapies have therefore emerged as promising alternatives for improving functional outcomes in patients with extensive burns^8^. Increasing evidence supports the use of mesenchymal stem cells (MSCs) to promote wound healing^8,9^. MSCs can migrate to injury sites, differentiate into multiple lineages, and are readily isolated and expanded in vitro^10^. Endogenous MSCs contribute to immunomodulation, angiogenesis, and tissue regeneration throughout all phases of burn healing^11,12^. During early inflammation, they modulate immune responses and promote neovascularisation^13^. Moreover, MSCs secrete paracrine factors that enhance extracellular-matrix synthesis, suppress inflammation, stimulate angiogenesis, and improve overall healing^14–16^. Initial approaches involving direct MSC transplantation were hindered by poor cell survival in the hostile burn microenvironment^17^ and concerns about potential tumorigenicity^18^. Interestingly, conditioned medium (CM) derived from MSCs exerts biological effects comparable to the cells themselves^19^, stimulating growing interest in MSC-CM and extracellular vesicles as safer, cell-free alternatives. These products are rich in paracrine mediators that modulate inflammation, promote granulation-tissue formation, enhance keratinocyte migration, and inhibit apoptosis, thereby accelerating wound repair^20–22^. Importantly, these acellular products exert their therapeutic effects through conserved bioactive factors, which may reduce immunogenicity and improve translational applicability compared with direct cell transplantation, particularly in cross-species experimental settings.

Previous studies have mainly investigated adipose-derived (AD-MSCs), bone-marrow-derived (BM-MSCs), and umbilical-cord-derived (UC-MSCs) MSCs, each offering distinct advantages for burn therapy^23^. BM-MSCs were among the first used clinically, suggesting improved angiogenesis and reduced scarring in patients with major burns^24,25^. Under inflammatory conditions, they secrete multiple growth factors—including TGF-β1, EGF, VEGF, PDGF, KGF, and FGF^26^—and their exosomes can enhance keratinocyte proliferation via inhibition of TGF-β/Smad signalling^27^. However, their invasive harvest procedures and limited clinical data restrict broader use. AD-MSCs display strong potential in skin regeneration by inhibiting TGF-β1-induced myofibroblast differentiation and reducing hypertrophic scarring^11^. They respond to burn exudates by increasing secretion of VEGF and IL-6, thereby enhancing vascularisation and healing^28–32^. Some studies indicate that AD-MSCs may outperform BM-MSCs in promoting angiogenesis and antifibrotic effects^33–35^, though clinical confirmation remains limited. UC-MSCs, in contrast, offer low immunogenicity, robust immunomodulatory capacity, abundant supply, and ethical advantages^36^. They have shown efficacy in diabetic-wound models by reducing oxidative stress and promoting angiogenesis^37^, and can stimulate dermal fibroblasts to secrete nerve growth factor, potentially ameliorating healing impairments related to denervation^38^. Human gingival tissue exhibits rapid healing with minimal scarring and inflammation^39,40^. Gingiva-derived MSCs (GMSCs), obtained from this microbially rich environment, possess bacterial resistance and potent immunomodulatory activity. They regulate innate immune cells—including macrophages, dendritic cells, and mast cells—and suppress pro-inflammatory cytokine secretion (e.g., TNF-α, IL-6)^41^. Although initially valued for their multipotency, the therapeutic benefits of MSCs are now attributed largely to their immunomodulatory and paracrine actions^42^, positioning GMSCs as promising candidates for burn repair. Notably, emerging evidence indicates that the GMSC secretome contains angiogenic and anti-inflammatory mediators such as vascular endothelial growth factor (VEGF), hepatocyte growth factor (HGF), and interleukin-10 (IL-10), which are critical for tissue repair and immune regulation^43^. Notably, MSC function can be enhanced through hormesis—a biphasic response in which mild stressors trigger adaptive benefits^44,45^. Preconditioning with inflammatory cytokines (e.g., IL-1β, TNF-α) or hypoxia has been shown to enhance MSC therapeutic potential^46,47^. Hypoxic preconditioning augments the paracrine activity of AD-MSCs, increasing secretion of VEGF and angiogenin^48^, while cytokine pretreatment boosts immunosuppressive capacity^49^. Mechanistically, hypoxia activates hypoxia-inducible factor-1α (HIF-1α), which regulates the expression of genes involved in angiogenesis, cell survival, and metabolic adaptation, thereby enhancing the regenerative potential of MSC-derived secretomes^50^. IL-1β-preconditioned GMSCs accelerate wound closure and reduce inflammation in vivo^51^, and hypoxia-treated MSCs improve graft survival^52^. These studies support the regenerative and immunomodulatory potential of GMSC-based therapies; however, the therapeutic efficacy and mechanistic effects of hypoxia-preconditioned GMSC-derived conditioned medium in full-thickness burn repair remain insufficiently characterized^51,52^. However, the effects of hypoxia-preconditioned GMSCs on burn-wound inflammation, graft integration, and scarring remain unexplored and warrant further investigation.

Taken together, modern burn care has evolved from survival-oriented to recovery-focused, emphasising rapid healing, high-quality scar formation, and functional restoration. Stem-cell-based therapies, particularly MSC-derived approaches, hold great promise by promoting angiogenesis and reducing fibrosis. Hormetic preconditioning provides a compelling strategy to enhance MSC efficacy. While hypoxia-preconditioned MSC-derived CM from adipose tissue or bone marrow has been investigated in wound models, no previous study has systematically evaluated hypoxia-enhanced GMSC-derived CM in the context of full-thickness thermal burns, nor its concurrent effects on keratinocytes and macrophages. Furthermore, the potential application of conditioned medium in combination with existing clinical strategies—such as skin grafting or biomaterial-based scaffolds—may further enhance therapeutic outcomes and warrants future investigation. Given the unique immunomodulatory profile and clinical accessibility of GMSCs, clarifying their paracrine actions in burn repair is of particular translational interest.

## Materials and methods

### Isolation and characterization of gingiva-derived mesenchymal stem cells (GMSCs)

Gingival tissues were collected from healthy donors undergoing orthodontic tooth extraction after obtaining written informed consent and approval from the Institutional Ethics Committee of Cheeloo College of Medicine, Shandong University, Shandong, China (Approval No. CCM-HT57-24). All procedures were performed in accordance with the ethical standards of the Declaration of Helsinki. Tissue samples were rinsed with PBS containing 1% penicillin–streptomycin (Beyotime, Shanghai, China), minced into approximately 1 mm³ fragments, and cultured in α-MEM medium (Gibco, Grand Island, NY, USA) supplemented with 10% FBS (Punosai, Shanghai, China) and 1% antibiotics. Explants were incubated at 37 °C in a humidified atmosphere with 5% CO₂. When cultures reached 80–90% confluence, cells were detached using 0.25% trypsin (Biosharp) and passaged; passages 3–5 were used for experiments. Passage 5 cells were selected because GMSCs maintained stable spindle-shaped morphology, MSC-associated marker expression, and differentiation capacity throughout passages 3–5 under our culture conditions. GMSCs were identified by immunofluorescence staining using antibodies against CD90 and CD105 (Proteintech, Wuhan, China), with HaCaT cells serving as negative controls. Cells were fixed with 4% paraformaldehyde (Servicebio, Wuhan, China), permeabilized with 0.5% Triton X-100 (Beyotime), and blocked with 10% goat serum. After incubation with primary and fluorophore-conjugated secondary antibodies, nuclei were counterstained with DAPI and imaged using a fluorescence microscope. Merged fluorescence images (CD90/DAPI and CD105/DAPI) were generated to confirm co-localization of marker expression.

To evaluate multipotency, GMSCs were induced toward osteogenic or adipogenic differentiation. For osteogenesis, cells were cultured for 2–3 weeks in DMEM (VivaCell, Shanghai, China) supplemented with 10% FBS, 100 nM dexamethasone, 10 mM β-glycerophosphate, and 50 µg/mL ascorbic acid. Mineralized nodules were visualized using Alizarin Red S (Solarbio, Beijing, China). For adipogenesis, cells were incubated for 2–3 weeks in induction medium containing 0.5 mM IBMX, 250 nM dexamethasone, 1 µg/mL insulin, and 10% FBS, followed by Oil Red O staining (Servicebio) to detect lipid droplets. All experiments were performed in triplicate. Chondrogenic differentiation was not performed because the present study focused on cutaneous wound healing rather than cartilage regeneration. We acknowledge that full trilineage differentiation and flow cytometric characterization of MSC surface markers would provide more comprehensive validation in accordance with established criteria; therefore, this has been included as a limitation of the study. Although flow-cytometric immunophenotyping and chondrogenic differentiation were not performed, the observed morphology, positive MSC-marker expression, and retained osteogenic/adipogenic differentiation potential supported preservation of mesenchymal stem-cell characteristics at passage 5.

#### Preparation of normoxic and hypoxia-preconditioned conditioned media (NM-GMSC-CM and HP-GMSC-CM)

When GMSCs reached approximately 80% confluence, the medium was replaced with serum-free α-MEM. Cells were seeded at a consistent density (~ 1 × 10⁶ cells per T75 flask) to standardize conditioned medium production. Cells were incubated either under normoxic conditions (21% O₂, 5% CO₂) or in a hypoxia chamber (Thermo Fisher Scientific, Waltham, MA, USA) at 1% O₂, 5% CO₂, and 94% N₂ for varying durations (6, 12, or 24 h). The collected supernatants were centrifuged at 1,000 × g for 5 min (Heraeus, Germany) and filtered through 0.22 μm membranes (Millipore, Burlington, MA, USA). Protein concentrations were determined by BCA assay to identify the optimal preconditioning time. CM from 12 h of hypoxia contained the highest protein concentration and migration-promoting activity; thus, 12 h was selected for all subsequent experiments. The resulting media were designated NM-GMSC-CM (normoxia-conditioned medium) and HP-GMSC-CM (hypoxia-preconditioned conditioned medium). Aliquots were stored at − 20 °C and thawed immediately before use. For in-vitro assays, both media were mixed 1:1 with complete culture medium.

#### Cell scratch assay

HaCaT human keratinocyte cells (N.E. Fusenig, Deutsches Krebsforschungszentrum, Heidelberg, Germany) were seeded into six-well plates and cultured until ~ 90% confluence. A linear scratch was created using a sterile 200 µL pipette tip. Detached cells were washed away with PBS, and the monolayers were treated with control medium, NM-GMSC-CM, or HP-GMSC-CM. Photographs of the wound area were taken at 0 h and 24 h using an inverted microscope (AxioVert.A1; Leica, Germany). Wound closure percentage was calculated using ImageJ software. All experiments were performed in triplicate (*n* = 3 independent experiments).

#### Transwell migration assay

Cell migration was analyzed using Transwell inserts (8 μm pores; Corning, NY, USA). HaCaT cells (1 × 10⁵ cells/well) in serum-free medium were placed in the upper chamber; the lower chamber contained control medium, NM-GMSC-CM, or HP-GMSC-CM. After 24 h of incubation, non-migrated cells were removed, and migrated cells on the lower surface were fixed with 4% paraformaldehyde and stained with 0.1% crystal violet (Servicebio, Wuhan, China). Five random fields per well were imaged under a light microscope, and the average cell counts were calculated. Each condition was tested in triplicate wells, and the experiment was repeated independently three times.

#### Inflammatory activation of THP-1 cells and cytokine measurement

THP-1 human monocytic cells (American Type Culture Collection, ATCC; TIB-202, Manassas, VA, USA) (1 × 10⁶ cells/mL) were seeded into plates and differentiated into macrophages with 100 ng/mL phorbol 12-myristate 13-acetate (PMA; Sigma-Aldrich, USA) for 24 h at 37 °C. After washing with serum-free RPMI-1640 (VivaCell, Shanghai, China), cells were polarized to the M1 phenotype by treatment with 20 ng/mL IFN-γ (PeproTech, NJ, USA) and 100 ng/mL LPS (Sigma-Aldrich) for another 24 h. M1-polarized macrophages were then treated with control medium, NM-GMSC-CM, or HP-GMSC-CM for 24 h. Where indicated, the PI3K inhibitor LY294002 (10 µM; Selleck, USA) was added 1 h before CM treatment. After incubation, supernatants were collected, and the levels of TNF-α and IL-1β were measured using human ELISA kits (4ABio, Beijing, China) following the manufacturer’s protocols.

#### Quantitative real-time PCR (RT-qPCR)

Total RNA was extracted from HaCaT cells treated with control medium, NM-GMSC-CM, or HP-GMSC-CM using TRIzol reagent (Invitrogen, Carlsbad, CA, USA). RNA concentration and purity were determined with a NanoDrop 2000 spectrophotometer (Thermo Fisher Scientific). cDNA was synthesized using the Hifair^®^ III First Strand cDNA Synthesis Kit (Yeasen, Shanghai, China). RT-qPCR was performed using SYBR Green Master Mix (Yeasen) on a StepOnePlus™ Real-Time PCR System (Applied Biosystems). GAPDH served as an internal reference. Primer sequences targeted LAMC2 and COL4A1. Relative expression was calculated using the 2^−ΔΔCt^ method. The PCR cycling conditions were as follows: initial denaturation at 95 °C for 5 min, followed by 40 cycles of denaturation at 95 °C for 10 s and annealing/extension at 60 °C for 30 s. All samples were analysed in triplicate. The primers used in this study are presented in Supplementary Table S1.

#### Western blot analysis

HaCaT cells and THP-1-derived M1 macrophages were treated with control medium, NM-GMSC-CM, HP-GMSC-CM, or HP-GMSC-CM plus LY294002 for 48 h. Cells were lysed in RIPA buffer containing protease and phosphatase inhibitors (Servicebio, Wuhan, China). Protein concentrations were measured using a BCA assay kit (Servicebio).

Equal protein amounts (30 µg) were separated on SDS-PAGE gels (Epizyme Biotech, Shanghai, China) and transferred to PVDF membranes (Millipore, Burlington, MA, USA). Membranes were blocked in 5% BSA for 1 h and incubated overnight at 4 °C with primary antibodies against p-AKT, total AKT, and β-actin/GAPDH (Abcam or Proteintech). After washing, membranes were incubated with HRP-conjugated secondary antibodies (Proteintech) for 1 h at room temperature. Bands were visualized by ECL (Thermo Fisher Scientific) and quantified with ImageJ.

#### Animal burn model and treatment

All animal procedures were approved by the Laboratory Animal Ethical and Welfare Committee of Shandong University Cheeloo College of Medicine (Approval No. DWLL-2024-273). All methods were carried out in accordance with relevant guidelines and regulations for the care and use of laboratory animals. This study is reported in accordance with the ARRIVE guidelines (https://arriveguidelines.org). Female C57BL/6J mice (8–10 weeks old, 20–25 g) were randomly divided into three groups (*n* = 6 per group): Control (PBS), NM (NM-GMSC-CM), and HP (HP-GMSC-CM). Mice were anesthetized with pentobarbital sodium (50 mg/kg, intraperitoneal injection) prior to burn induction. Depth of anesthesia was verified by loss of the pedal withdrawal reflex before the burn procedure. After dorsal shaving, a 2 cm² full-thickness burn wound was created using a thermostatic burn instrument equipped with a 1 cm diameter metal head preheated to 100 °C for 10 s. Twenty-four hours post-injury, 0.15 mL of PBS, NM-GMSC-CM, or HP-GMSC-CM was injected via multi-point intradermal/perilesional routes around the wound margin once daily for 7 days. Wounds were photographed on days 0, 4, 8, and 15 post-injury, and wound areas were quantified using ImageJ software. At the study endpoint (day 15), mice were euthanized under anesthesia by an overdose of pentobarbital sodium (150 mg/kg, intraperitoneal injection) followed by a secondary physical method (cervical dislocation) to ensure death. Death was confirmed by absence of respiration and heartbeat and lack of reflexes (e.g., pedal/corneal reflex) prior to tissue collection.

#### Histological and immunohistochemical (IHC) analyses

Tissues were fixed in 4% paraformaldehyde (Servicebio), embedded in paraffin, and sectioned (5 μm). Sections were stained with hematoxylin–eosin (H&E) for morphology and Masson’s trichrome for collagen deposition. For IHC, deparaffinized sections were subjected to antigen retrieval, blocked with normal serum, and incubated overnight at 4 °C with primary antibodies against pan-cytokeratin (Pan-CK) and tenascin-C (TNC) (Abcam). After incubation with HRP-conjugated secondary antibodies, signals were visualized using DAB, and nuclei were counterstained with hematoxylin. Stained areas were imaged under a light microscope, and Pan-CK and TNC positivity were quantified using ImageJ. For each animal, at least three non-overlapping tissue sections were analyzed, and five randomly selected microscopic fields from each section were quantified. The average optical density (AOD) for Pan-CK and TNC staining was calculated for each field, and the mean value from all analyzed fields was used to generate one biological replicate per animal. Group-level statistical comparisons were then performed using the animal-level mean values, rather than treating individual images or fields as independent replicates.

Detailed information regarding the reagents, antibodies, culture media, ELISA kits, inhibitors, and software used in this study, including vendor names and catalog numbers, is provided in Supplementary Table S2.

### Statistical analysis

All data are expressed as mean ± standard deviation (SD). Statistical analyses were performed using GraphPad Prism 9.0 (GraphPad Software, San Diego, CA, USA). Differences between two groups were evaluated with Student’s t-test. Multiple-group comparisons were analyzed using one-way ANOVA followed by Tukey’s post hoc test. Values of *p* < 0.05 were considered statistically significant. All experiments were performed with at least three independent biological replicates unless otherwise specified.

## Results

### Characterization of the stemness of GMSCs

**Fig. 1:**
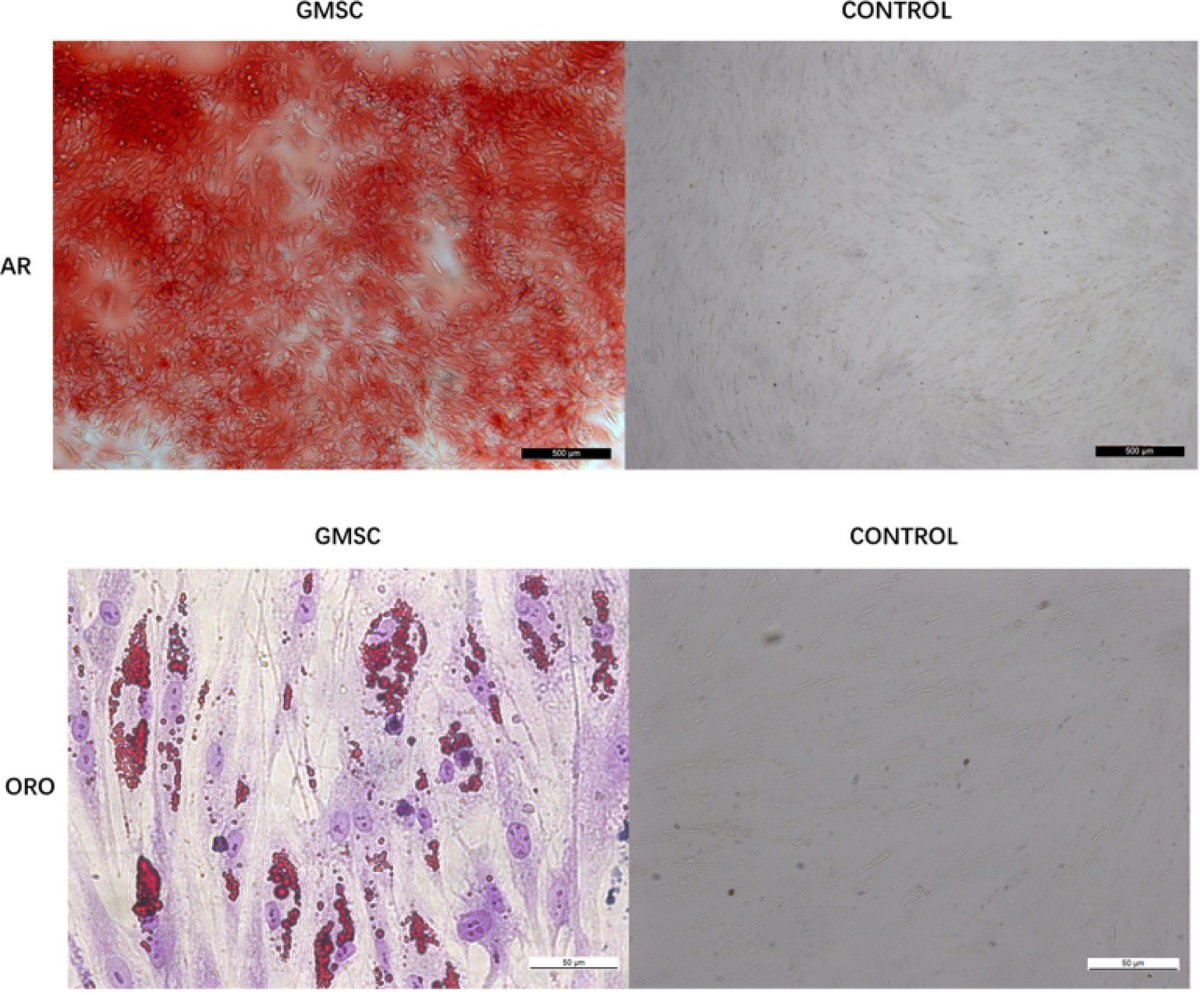

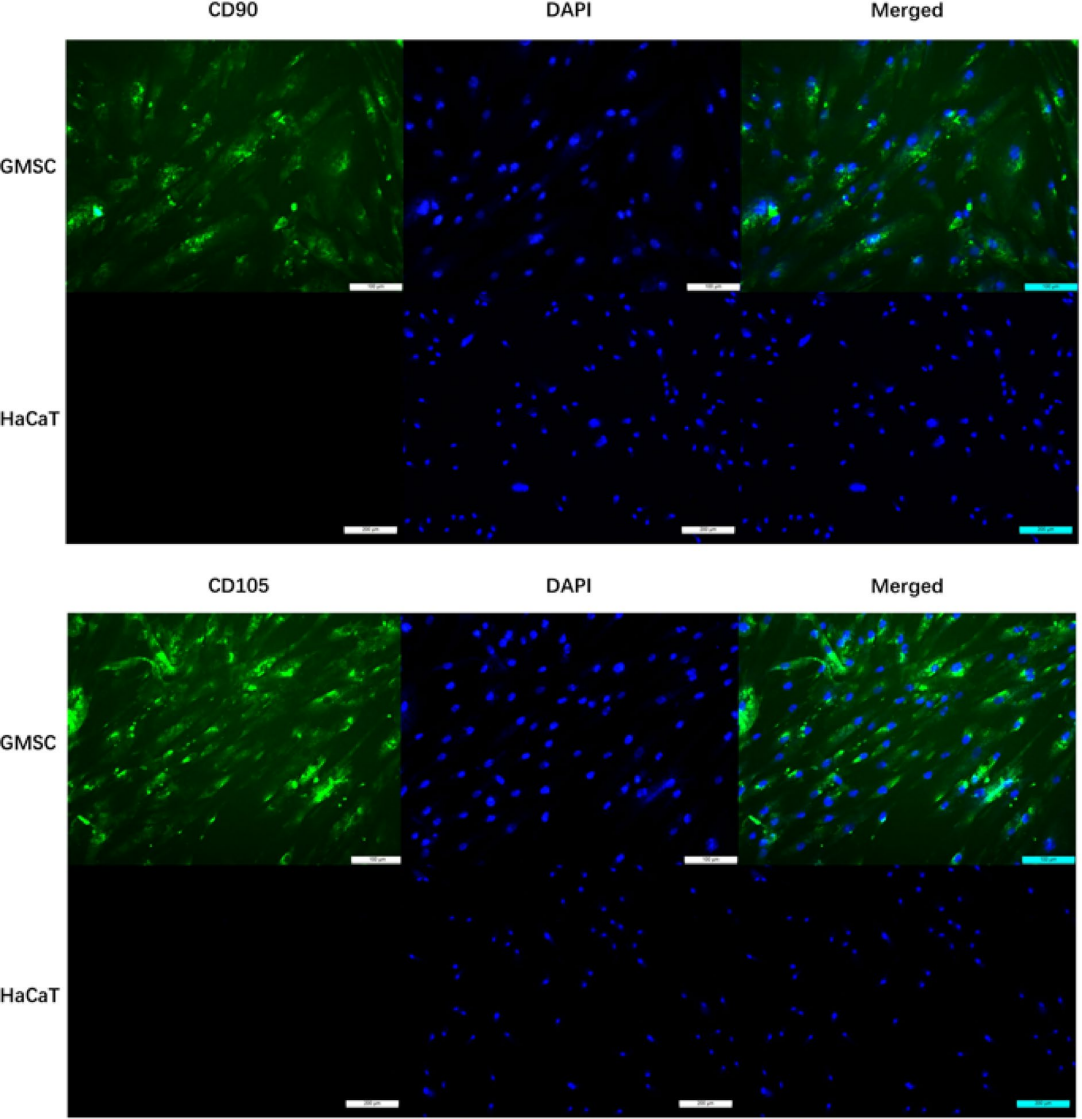
Characterization of gingiva-derived mesenchymal stem cells (GMSCs). (**A**) Alizarin Red S (ARS) staining showing abundant mineralized nodules in GMSCs following osteogenic induction compared with the unstimulated control. (**B**) Oil Red O (ORO) staining demonstrating intracellular lipid droplet accumulation in GMSCs after adipogenic induction, whereas control cells show no lipid deposition. (**C**) Immunofluorescence staining of CD90 (green), DAPI nuclear staining (blue), and corresponding merged images in GMSCs and HaCaT cells (negative control). (**D**) Immunofluorescence staining of CD105 (green), DAPI nuclear staining (blue), and corresponding merged images in GMSCs and HaCaT cells (negative control). Merged images confirm co-localization of mesenchymal markers with nuclei in GMSCs. Brightness and contrast were uniformly adjusted across all fluorescence images for clarity without altering the original data. Scale bars = 500 μm (**A**), 50 μm (**B**), and 100–200 μm (**C**–**D**).

GMSCs were successfully isolated from healthy human gingival tissues and exhibited a typical fibroblast-like morphology under light microscopy, characterized by spindle-shaped cell bodies and prominent nuclei. The cells adhered firmly to culture flasks and proliferated to form a homogeneous monolayer. To evaluate multipotency, GMSCs were subjected to osteogenic and adipogenic induction using specific differentiation media. After osteogenic induction, Alizarin Red S staining revealed prominent orange-red mineralized nodules (Fig. 1A). Following adipogenic induction, Oil Red O staining confirmed the presence of numerous intracellular red lipid droplets (Fig. 1B). Immunofluorescence staining demonstrated strong positive expression of MSC markers CD90 and CD105 in GMSCs, whereas HaCaT cells showed no detectable staining (Fig. 1C, D), confirming the mesenchymal stem cell phenotype. Merged fluorescence images (CD90/DAPI and CD105/DAPI) further confirmed co-localization of marker expression within GMSCs, supporting their mesenchymal identity.

### Optimization of hypoxic preconditioning and conditioned medium preparation

**Fig. 2:**
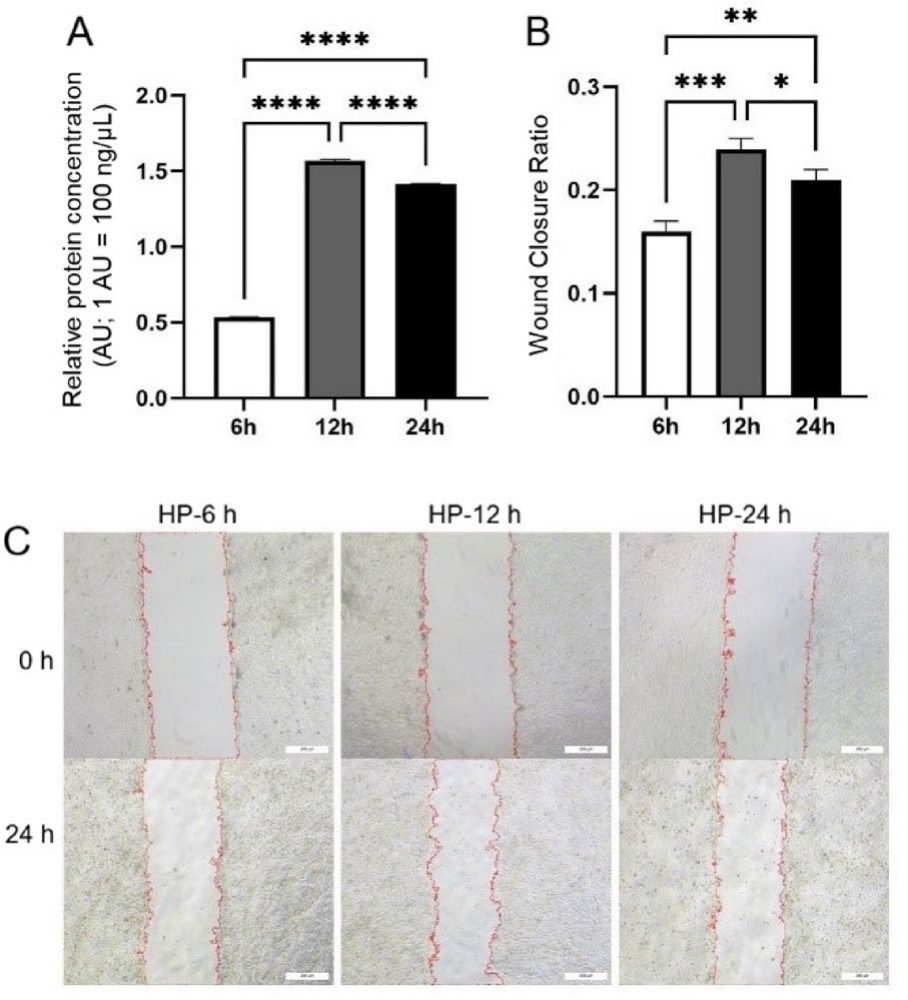
Effects of hypoxic preconditioning on protein secretion and migration of GMSCs. (**A**) Relative protein concentration in the conditioned medium (CM) of GMSCs after 6, 12, and 24 h of hypoxic preconditioning, measured by BCA assay. (**B**) Wound-closure ratio of HaCaT cells treated with CM from hypoxia-preconditioned GMSCs at different durations. (**C**) Representative wound-healing images of HaCaT cells at 0 h and 24 h after treatment with 6 h-, 12 h-, and 24 h-hypoxia CM. Scale bars = 200 μm. Data are expressed as mean ± SD; * *p* < 0.05, ** *p* < 0.01, *** *p* < 0.001, **** *p* < 0.0001.

To determine the optimal duration of hypoxic preconditioning, GMSCs were exposed to 1% O₂ for 6, 12, or 24 h, and the protein concentration and migration-promoting activity of the collected conditioned media (CM) were compared. BCA assay revealed that CM from GMSCs preconditioned for 12 h contained the highest protein concentration (Fig. 2A). In a scratch-wound assay using HaCaT cells, CM collected after 12 h of hypoxia produced the greatest enhancement in cell migration, whereas CM from 24 h of hypoxia showed diminished activity (Fig. 2B, C). Based on these findings, a 12-hour hypoxic preconditioning regimen was selected for subsequent preparation of HP-GMSC-CM. These results indicate that excessive hypoxic exposure may reduce the functional activity of the GMSC secretome, possibly due to cellular stress or altered secretion dynamics.

### HP-GMSC-CM enhances HaCaT cell migration

**Fig. 3:**
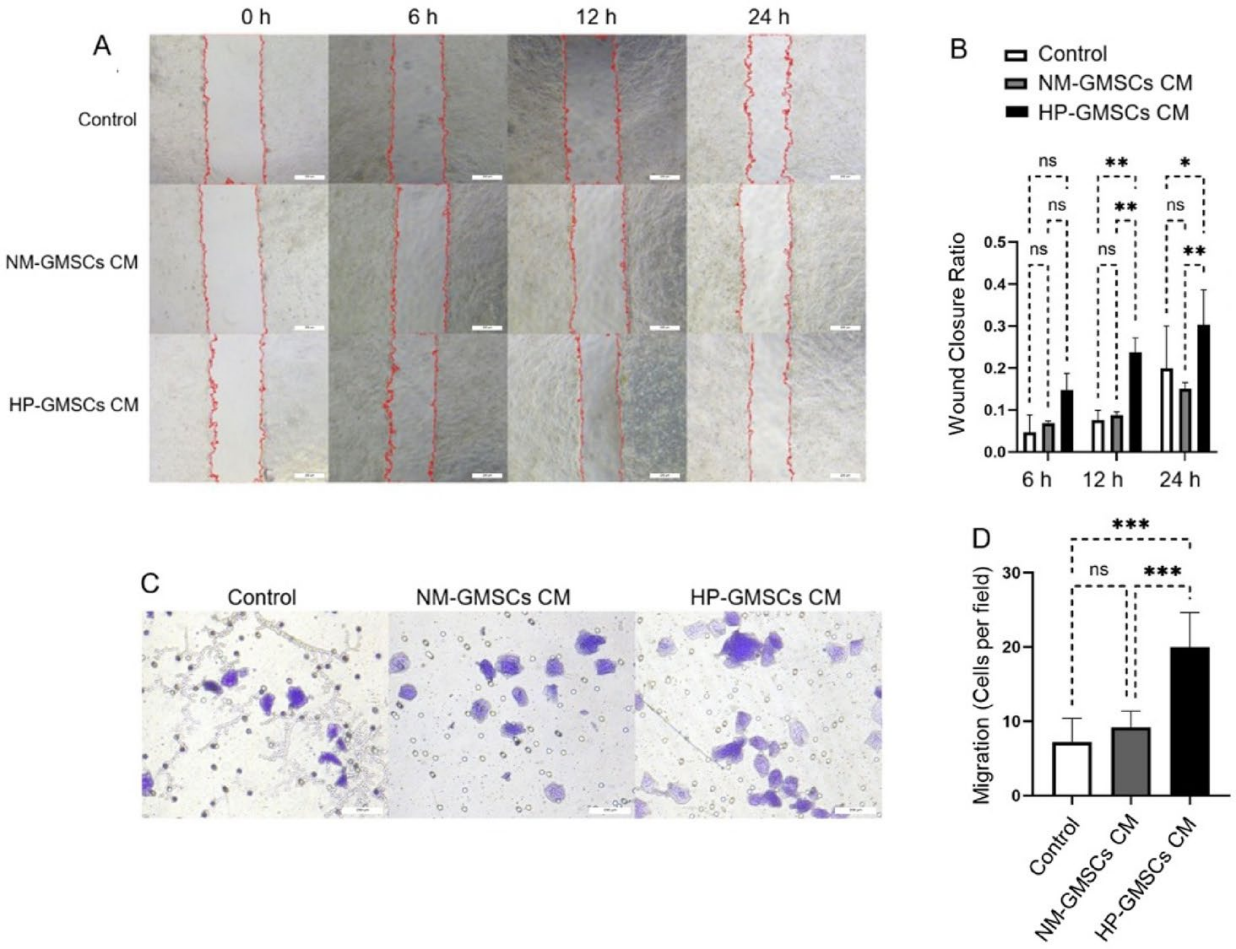
Effects of GMSC-derived conditioned medium on migration and wound healing in HaCaT cells. (**A**) Scratch-wound images of HaCaT cells treated with control medium, normoxia-conditioned medium (NM)-GMSCs CM, or hypoxia-preconditioned (HP)-GMSCs CM for 0–24 h. (**B**) Quantification of wound-closure ratio at 6, 12, and 24 h. (**C**) Transwell migration assay showing HaCaT cells after 24 h of treatment. (**D**) Quantification of migrated cells per field. Scale bars = 200 μm. Data are mean ± SD; ns = not significant, * *p* < 0.05, ** *p* < 0.01, *** *p* < 0.001, **** *p* < 0.0001.

The effects of NM-GMSC-CM and HP-GMSC-CM on HaCaT cell migration were assessed by scratch and Transwell assays. After 12 and 24 h of incubation, the wound-closure rate was significantly higher in the HP-GMSC-CM group than in both the NM-GMSC-CM and control groups (Fig. 3A, B). Consistently, Transwell assays demonstrated a marked increase in the number of migrating HaCaT cells in the HP-GMSC-CM group compared with the other two groups (Fig. 3C, D). Quantitative analysis was performed from at least five randomly selected fields per well across three independent experiments. These results indicate that hypoxic preconditioning enhances the paracrine profile of GMSCs, thereby markedly promoting keratinocyte migration.

### HP-GMSC-CM upregulates DEJ proteins and attenuates inflammation

**Fig. 4:**
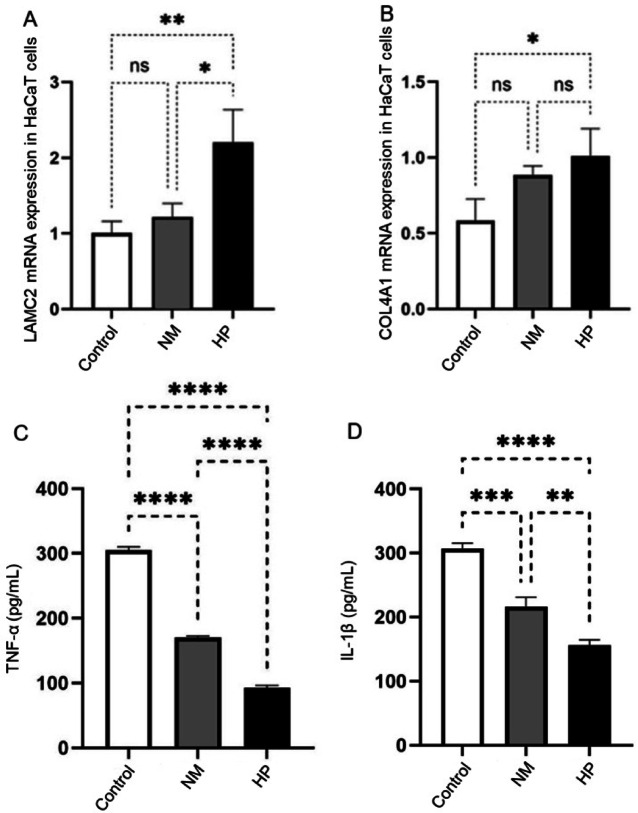
Expression of extracellular matrix and inflammatory genes in HaCaT cells treated with GMSC-derived CM. (**A**) LAMC2 mRNA expression in HaCaT cells. (**B**) COL4A1 mRNA expression in HaCaT cells. (**C**) TNF-α and (**D**) IL-1β levels in M1-polarised THP-1 macrophage supernatants measured by ELISA. Data are mean ± SD; ns = not significant, * *p* < 0.05, ** *p* < 0.01, *** *p* < 0.001, **** *p* < 0.0001.

Proteins associated with the dermal–epidermal junction (DEJ) are vital for maintaining skin integrity and elasticity during wound repair. In the process of healing, granulation tissue is gradually replaced by collagen and elastin fibers secreted by epithelial cells, leading to tissue remodeling^52^. DEJ components contribute to multiple phases of skin regeneration—from angiogenesis to re-epithelialization and are key constituents of clinical skin scaffolds^53^. To examine the impact of HP-GMSC-CM on DEJ-related proteins, HaCaT cells were treated with HP-GMSC-CM or NM-GMSC-CM for 48 h, with HaCaT self-conditioned medium as a control. RT-qPCR analysis revealed that mRNA expression of LAMC2 (laminin γ2 chain) and COL4A1 (type IV collagen α1 chain) was significantly upregulated in HaCaT cells cultured with HP-GMSC-CM compared with those treated with NM-GMSC-CM (Fig. 4A, B). These results suggest that hypoxia enhances the ability of GMSCs to promote extracellular-matrix remodeling in epidermal cells through upregulation of key DEJ proteins.

Timely regulation of inflammation is also critical for successful wound repair. Among immune cells, macrophages play an important role in orchestrating the burn microenvironment. In this study, THP-1-derived macrophages were differentiated with PMA and polarized toward the M1 phenotype using LPS and IFN-γ. ELISA of M1 macrophage supernatants showed that co-culture with HP-GMSC-CM significantly reduced secretion of TNF-α and IL-1β compared with both NM-GMSC-CM and control medium (Fig. 4C, D). These findings indicate that HP-GMSC-CM more effectively suppresses M1 macrophage activation and pro-inflammatory cytokine release, thereby attenuating excessive inflammation and promoting its resolution. Collectively, these results demonstrate that HP-GMSC-CM facilitates wound healing through a dual mechanism: (i) reinforcing DEJ stability by upregulating LAMC2 and COL4A1, and (ii) modulating innate immune responses to limit inflammatory injury and foster regeneration.

### HP-GMSC-CM promotes burn wound healing in vivo

**Fig. 5:**
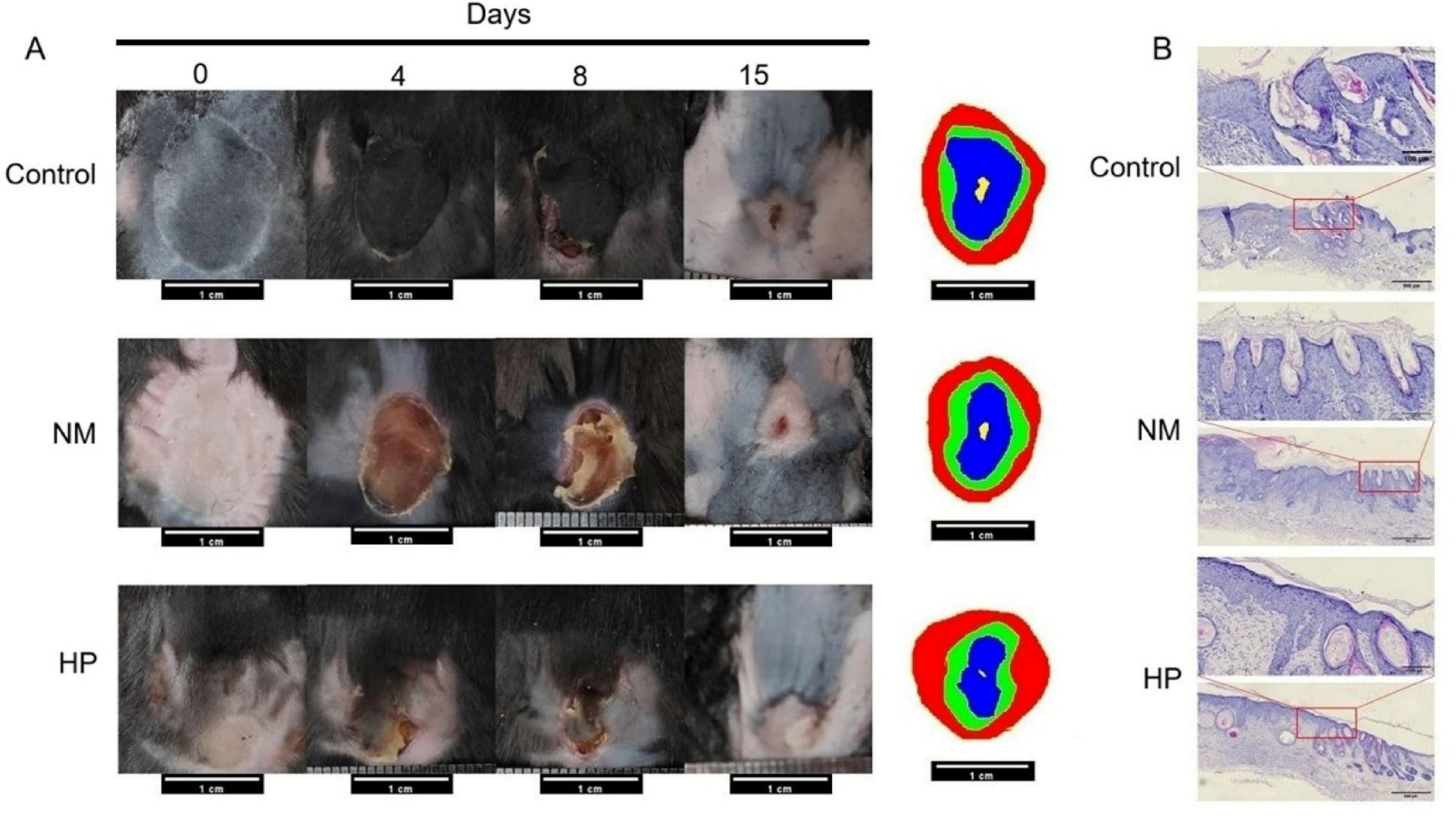
Effect of GMSC-derived conditioned medium on burn-wound healing in vivo. (**A**) Representative photographs of burn wounds in control, NM-, and HP-GMSC-treated mice on days 0, 4, 8, and 15, with quantitative segmentation masks showing wound-healing progression. (**B**) Hematoxylin-and-eosin (H&E)-stained skin sections illustrating epithelial regeneration and dermal remodeling in each group. Scale bars = 1 cm (**A**) and 100 μm (**B**).

**Fig. 6:**
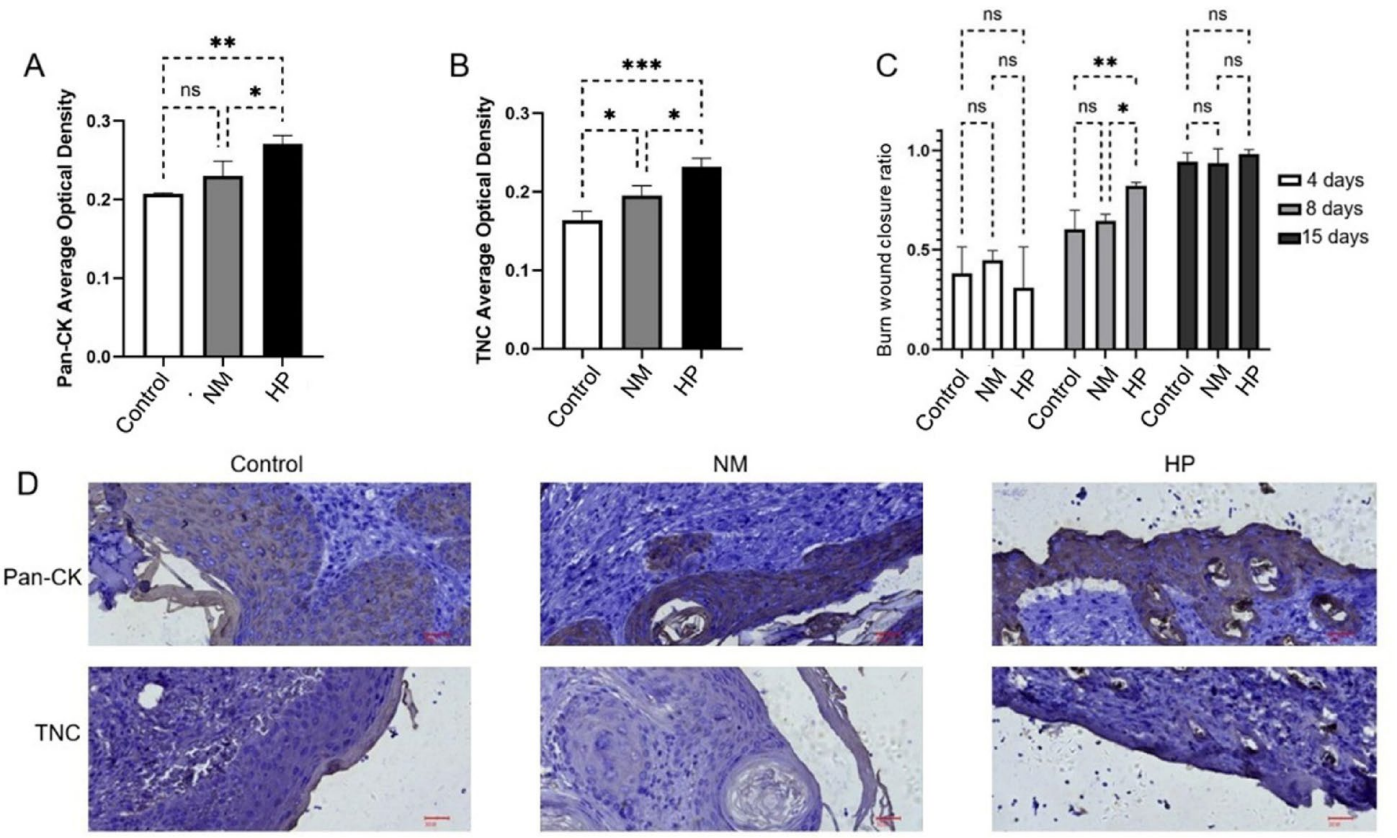
Expression of Pan-CK and Tenascin C (TNC) in regenerated skin tissue after treatment. (**A**) Average optical density (AOD) of Pan-CK staining. (**B**) Average optical density (AOD) of TNC staining. (**C**) Quantitative analysis of wound closure ratios at days 4, 8, and 15. (**D**) Representative immunohistochemical images of Pan-CK and TNC expression in regenerated skin from the control, NM-, and HP-treated groups. Scale bars = 100 μm. Data are mean ± SD; ns = not significant, * *p* < 0.05, ** *p* < 0.01, *** *p* < 0.001.

To verify the in vitro findings, a full-thickness skin-burn model was established in C57BL/6J mice. A standardized circular wound (~ 2 cm²) was created using a thermostatic burn instrument. Twenty-four hours after injury, 0.15 mL of PBS, NM-GMSC-CM, or HP-GMSC-CM was administered via multi-point intradermal injections around the wound periphery. Wounds were photographed and measured on days 0, 4, 8, and 15. Macroscopic observations revealed accelerated wound closure in the HP-GMSC-CM group compared with NM-GMSC-CM and PBS controls (Fig. 5A). Quantitative analysis confirmed that the wound-closure rate was significantly higher in the HP-GMSC-CM group, particularly by day 8 (Fig. 6C). Histopathological examination on day 15 showed dense inflammatory-cell infiltration and extensive granulation tissue in PBS-treated wounds, with aberrant follicular structures in some regions (Fig. 5B). In contrast, HP-GMSC-CM-treated wounds exhibited nearly complete restoration of dermal–epidermal architecture with well-organized collagen deposition. Immunohistochemical staining further demonstrated stronger Pan-CK expression, reflecting enhanced epithelial regeneration, and a larger TNC-positive area—an extracellular-matrix component essential for restoring skin mechanical strength (Fig. 6A, B, D). Quantification of immunostaining was performed using ImageJ-based analysis of average optical density (AOD) across multiple sections per sample. Together, these data confirm the superior in vivo wound-healing efficacy of HP-GMSC-CM.

### HP-GMSC-CM promotes wound healing via the PI3K/AKT pathway

**Fig. 7:**
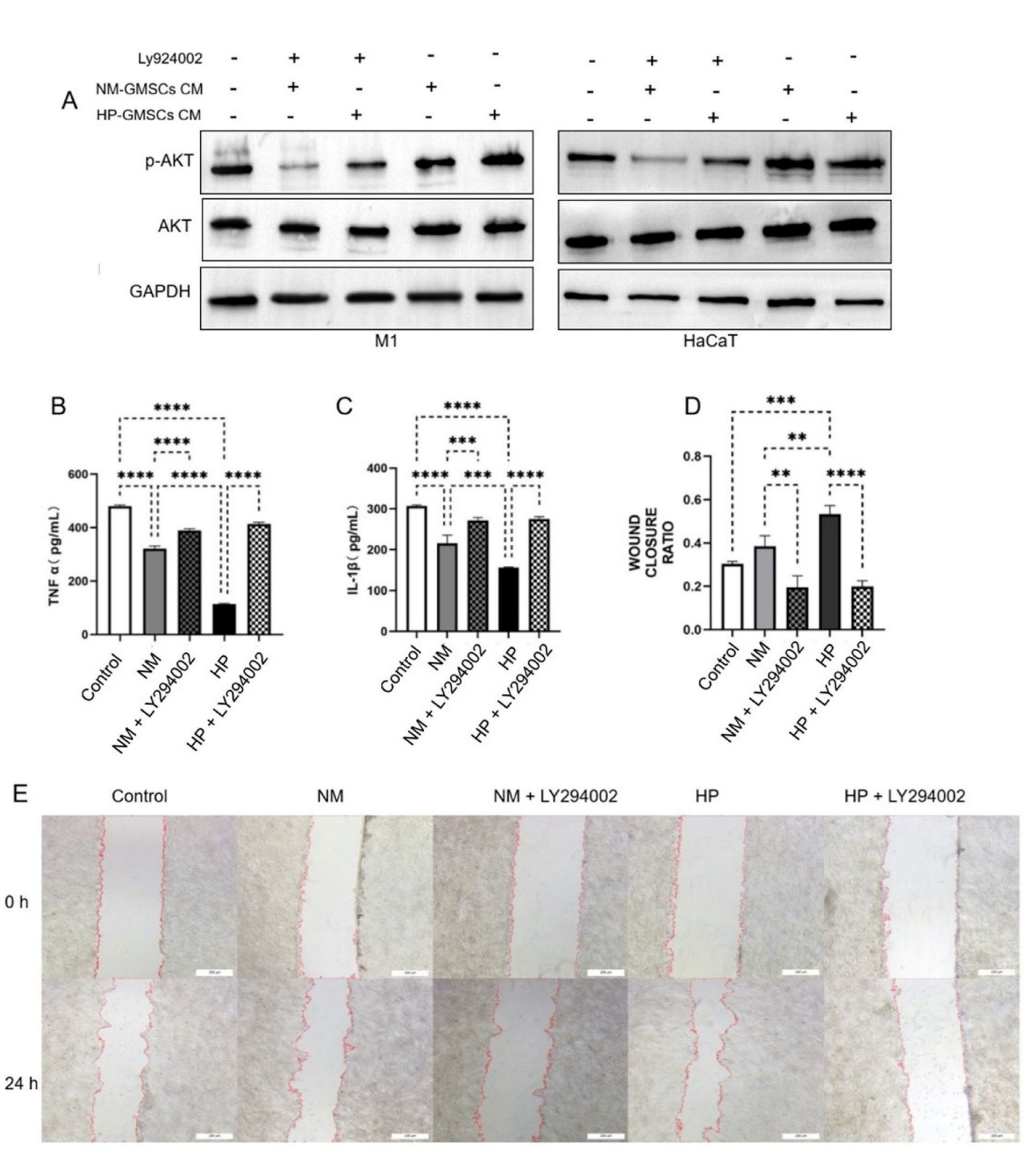
PI3K/AKT signaling is involved in the anti-inflammatory and pro-migratory effects of HP-GMSCs CM. (**A**) Western-blot analysis of phosphorylated AKT (p-AKT), total AKT, and GAPDH in M1 macrophages and HaCaT cells treated with NM-GMSCs CM or HP-GMSCs CM, with or without the PI3K inhibitor LY294002. (**B**, **C**) ELISA quantification of TNF-α and IL-1β levels. (**D**) Wound-closure ratio of HaCaT cells after 24 h. (**E**) Representative wound-healing images (0 h and 24 h) under the same treatment conditions. Scale bars = 200 μm. Data are mean ± SD; ** *p* < 0.01, *** *p* < 0.001, **** *p* < 0.0001.

Although HP-GMSC-CM demonstrated enhanced wound-healing efficacy, the **underlying** molecular mechanism remained unclear. The PI3K/AKT pathway is known to regulate tissue repair, regeneration, and re-epithelialization^54–56^. We therefore investigated whether HP-GMSC-CM exerts its therapeutic effects through this pathway. Western-blot analysis revealed increased phosphorylation of AKT (p-AKT) in HaCaT cells and M1 macrophages treated with NM-GMSC-CM or HP-GMSC-CM compared with control medium. Pretreatment with the PI3K inhibitor LY294002 partially attenuated p-AKT expression while total AKT levels remained relatively stable (Fig. 7A). Residual p-AKT expression remained detectable following LY294002 treatment, suggesting partial rather than complete inhibition of the pathway under the present experimental conditions. Functional assays further demonstrated that inhibition of PI3K/AKT signaling attenuated the pro-healing effects of HP-GMSC-CM. In scratch assays, addition of LY294002 significantly reduced the wound-closure rate of HaCaT cells treated with HP-GMSC-CM (Fig. 7D, E). Similarly, in the M1 macrophage model, LY294002 reversed the HP-GMSC-CM-mediated suppression of TNF-α and IL-1β, resulting in increased cytokine levels compared with HP-GMSC-CM treatment alone (Fig. 7B, C). Collectively, these findings support the involvement of PI3K/AKT signaling in mediating the pro-regenerative and anti-inflammatory effects of GMSC-derived conditioned medium. Specifically, HP-GMSC-CM appears to promote wound repair, at least in part, through activation of PI3K/AKT-associated signaling cascades in epithelial and immune cells.

## Discussion

Mesenchymal stem cells (MSCs) have attracted considerable attention for their therapeutic potential in burn wound healing. Although bone-marrow-derived MSCs (BM-MSCs) have been extensively studied and shown to accelerate wound closure and promote angiogenesis without increasing infection risk^24,25^, their clinical use remains limited by invasive harvesting procedures and relatively scarce clinical evidence. In contrast, gingiva-derived MSCs (GMSCs) offer distinct advantages, including greater accessibility, intrinsic rapid-healing capacity, and reduced inflammatory reactivity in a bacteria-rich oral environment^39,40^. GMSCs also exhibit potent immunomodulatory effects on innate immune cells—including macrophages, dendritic cells, and mast cells—further enhancing their therapeutic utility^41,42^. Nevertheless, the precise mechanisms by which GMSCs facilitate burn-wound repair remain incompletely understood. Conventional stem-cell therapies relying on direct cell transplantation face obstacles such as poor cell survival and potential tumorigenicity^17,18^. Increasing evidence indicates that the regenerative effects of MSCs are largely mediated through paracrine mechanisms^19^. Conditioned medium (CM) collected from MSC cultures represents a promising cell-free therapeutic approach, containing a wide spectrum of bioactive molecules—including extracellular vesicles, soluble proteins, cytokines, growth factors, and metabolites—that act synergistically to promote tissue repair^57–59^. These bioactive components, including vascular endothelial growth factor (VEGF), hepatocyte growth factor (HGF), and anti-inflammatory cytokines such as interleukin-10 (IL-10), have been reported to play important roles in angiogenesis, immune modulation, and tissue regeneration^43^. Compared with exosome-based therapies, CM demonstrates comparable or superior efficacy in enhancing cell proliferation and tissue regeneration while eliminating the risks associated with live-cell administration^60,61^. Importantly, as an acellular therapeutic product, CM reduces the risk of immunogenicity, making it particularly suitable for xenogeneic applications in preclinical models and supporting its translational potential.

The present study demonstrates that hypoxia-preconditioned GMSC-conditioned medium (HP-GMSC-CM) effectively enhances burn-wound healing both in vitro and in vivo. Previous studies have demonstrated therapeutic effects of MSC-derived conditioned medium in wound repair, including hypoxia-preconditioned bone marrow MSC-conditioned medium^20^. However, the present study differs in specifically evaluating hypoxia-preconditioned gingiva-derived MSC-conditioned medium in a full-thickness burn model while simultaneously examining keratinocyte migration, macrophage-mediated inflammation, and PI3K/AKT-associated signaling responses. MSCs typically remain quiescent until exposed to injury or inflammatory stimuli^43^. Preconditioning with hypoxia or inflammatory cytokines can substantially augment MSC therapeutic efficacy^46,47^. Mechanistically, hypoxic preconditioning is known to activate hypoxia-inducible factor-1α (HIF-1α), which upregulates genes involved in angiogenesis, cell survival, and metabolic adaptation, thereby enhancing the secretion of regenerative and immunomodulatory factors^48,62^. The current results provide the first evidence that HP-GMSC-CM not only promotes burn-wound closure but also attenuates inflammation, likely through activation of the PI3K/AKT signalling pathway. Prolonged hypoxic exposure, however, may exert detrimental effects such as reduced cellular function and CM potency, possibly due to excessive cellular stress and accumulation of toxic mediators. In our optimisation experiments, a 12-hour hypoxic preconditioning period at 1% O₂ achieved the optimal balance between protein content and functional activity, whereas 24-hour exposure diminished the pro-migratory effects of CM. Although these conditions were based on established protocols^63,64^, the optimal oxygen tension and duration for GMSC preconditioning warrant further investigation. Future studies may explore advanced optimization strategies, including three-dimensional culture systems, biomaterial-assisted conditioning, or cytokine priming, to further enhance the therapeutic potency of the GMSC secretome.

Excessive inflammation during the early post-burn phase can impede healing and lead to chronic, non-healing wounds^65^. Macrophages play a pivotal role in orchestrating this immune response. Previous studies have shown that MSCs promote macrophage polarisation from a pro-inflammatory M1 phenotype toward an anti-inflammatory M2 phenotype, partly through modulation of PI3K/AKT signalling^66–68^. Consistent with these findings, our results indicate that HP-GMSC-CM more effectively suppresses M1 macrophage activation and inflammatory cytokine release than normoxia-derived GMSC-CM (NM-GMSC-CM), thereby facilitating inflammation resolution and tissue repair. The relatively modest effects observed with NM-GMSC-CM in some assays may reflect insufficient secretion of bioactive mediators under physiological oxygen conditions, underscoring the critical role of hypoxic preconditioning in enhancing the GMSC secretome and overall therapeutic efficacy. Although angiogenesis was not directly evaluated in the present study, the observed improvements in wound closure, extracellular matrix remodeling, and epithelial regeneration suggest that enhanced vascularization may contribute to the therapeutic effects of HP-GMSC-CM. This study expands current understanding of GMSC-based regenerative therapy by providing the first experimental evidence that hypoxia-preconditioned GMSC-derived CM accelerates full-thickness burn-wound healing through coordinated modulation of epidermal and immune cell responses. The findings reveal that HP-GMSC-CM enhances keratinocyte migration, upregulates dermal-epidermal junction (DEJ)-related genes (LAMC2 and COL4A1), and attenuates macrophage-mediated inflammation, accompanied by activation of PI3K/AKT signalling. These results highlight a clinically translatable, cell-free approach leveraging simple hypoxic preconditioning to augment the therapeutic potency of GMSC secretome for cutaneous regeneration. Furthermore, integration of HP-GMSC-CM with existing clinical strategies, such as skin grafting or biomaterial-based scaffolds, may further enhance therapeutic outcomes and represents a promising direction for future translational research. In addition, alternative formulations, including exosome-enriched fractions or concentrated secretome extracts, may provide comparable or enhanced therapeutic benefits and warrant further investigation.

This study has several limitations. First, activation of the PI3K/AKT pathway was demonstrated only in vitro, and no in vivo verification or downstream target analysis was performed; therefore, the mechanistic link should be interpreted as associative rather than causal. Second, the conditioned medium was not further characterized to identify specific bioactive components responsible for the observed effects, and no proteomic or cytokine profiling was performed. Third, angiogenesis-specific functional assays were not conducted, limiting direct assessment of vascular contributions to wound healing. Fourth, a xenogeneic model using human-derived GMSCs in mice was employed, which, although widely used in preclinical studies, may not fully recapitulate immune interactions in human clinical settings. Fifth, flow cytometric characterization of MSC surface markers was not performed, and immunofluorescence-based validation was used instead. Sixth, only HaCaT keratinocytes and THP-1-derived macrophages were used as in vitro models, which may not fully represent primary human cell behavior. In addition, densitometric quantification of western blot bands was not performed, which may limit precise assessment of pathway inhibition. Finally, long-term safety and efficacy were not evaluated in this study. Despite these limitations, the findings provide a solid experimental foundation supporting the therapeutic potential of hypoxia-preconditioned GMSC-derived conditioned medium in promoting burn-wound repair.

## Conclusion

This study provides novel insights into the therapeutic potential and underlying mechanisms through which hypoxia-preconditioned gingival mesenchymal stem cell-conditioned medium (HP-GMSC-CM) facilitates burn-wound healing. HP-GMSC-CM promoted keratinocyte migration, modulated inflammatory cytokine release, and improved burn-wound repair, accompanied by activation of PI3K/AKT signalling in keratinocytes and macrophages. These data support PI3K/AKT signalling as being involved in the observed effects, which warrants further investigation. Although the precise composition of the conditioned medium and its individual bioactive components remain to be elucidated, the present findings highlight the potential of hypoxia-preconditioned GMSC-derived secretome as a promising cell-free therapeutic strategy. Furthermore, validation in large-animal models that more closely recapitulate human skin physiology will be essential for establishing a solid experimental foundation for the clinical translation of this cell-free therapeutic strategy.

## Supplementary Information

Below is the link to the electronic supplementary material.

Supplementary Material 1

Supplementary Material 2

Supplementary Material 3

## Data Availability

The datasets generated and/or analyzed during the current study are available from the corresponding author on reasonable request.
