# Supplementary material for: Hypoxia-preconditioned gingiva-derived mesenchymal stem cell-conditioned medium accelerates burn wound healing
Source: Sci Rep. 2026 May 25;16:23486. doi: 10.1038/s41598-026-54462-8 (PMC13415783; doi:10.1038/s41598-026-54462-8)
Supplement: Supplementary file 1 — Supplementary Material 1 [file 41598_2026_54462_MOESM1_ESM.docx]

**Supplementary Table S1:** List of Primer Sequences used in the study

| **Primer** | **Sequences(5’-3’)** |
| --- | --- |
| LAMC2 | F: GGCTGGTCTTACTGGAGCAG |
|  | R: TATGGCAGCTTCACTGTTGC |
| COL4A1 | F: CTGGTCCAAGAGGATTTCCA |
|  | R: TCATTGCCTTGCACGTAGAG |
| GAPDH | F: GAAGGTGAAGGTCGGAGTC |
|  | R: GAAGATGGTGATGGGATTTC |
