# Supplementary figures and images for: Hypoxia-preconditioned gingiva-derived mesenchymal stem cell-conditioned medium accelerates burn wound healing
Source: Sci Rep. 2026 May 25;16:23486. doi: 10.1038/s41598-026-54462-8 (PMC13415783; doi:10.1038/s41598-026-54462-8)

M1

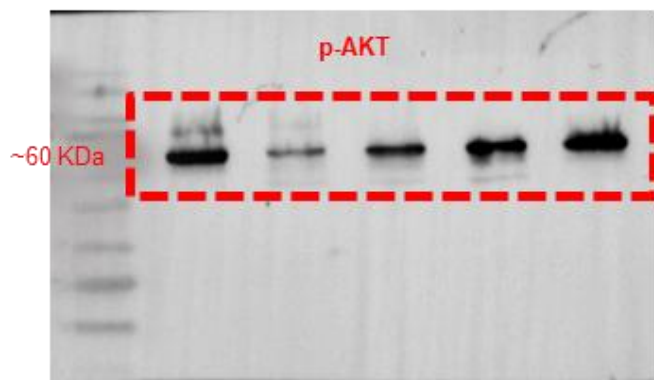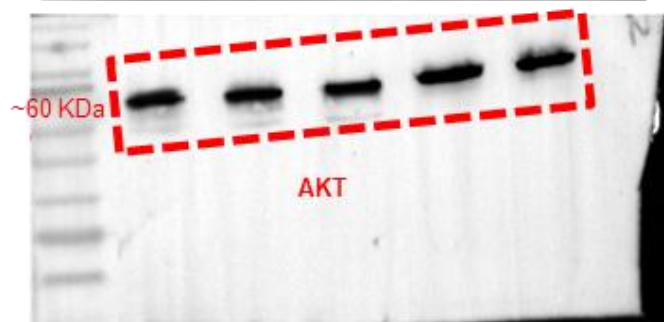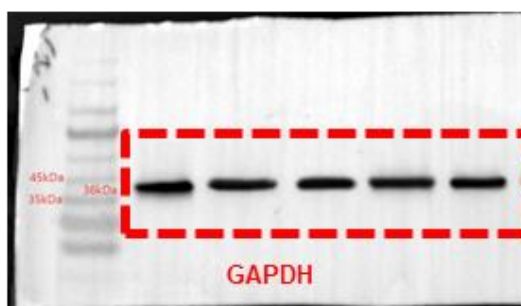

HaCaT

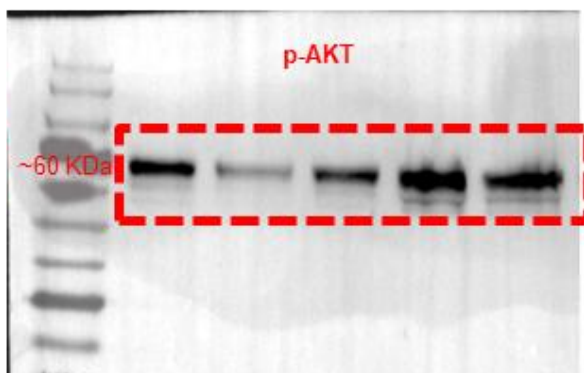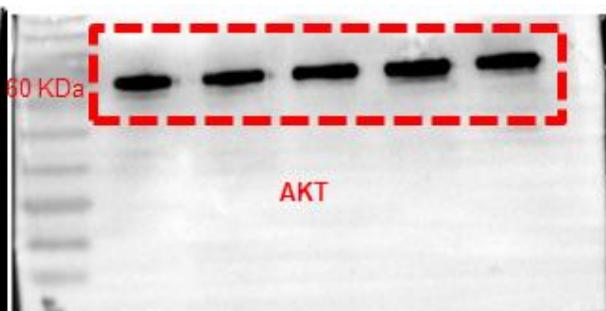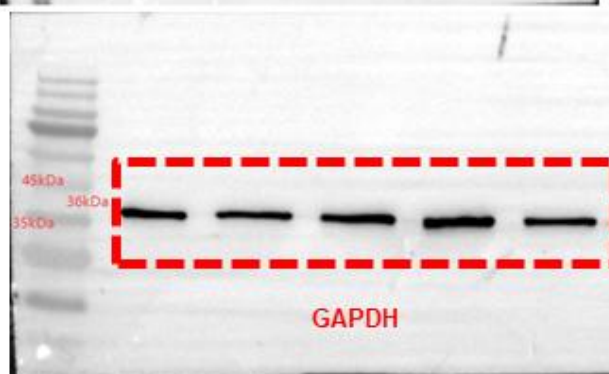

Supplement: Supplementary file 2 — Supplementary Material 2 [file 41598_2026_54462_MOESM2_ESM.pdf]
