# Supplementary material for: Hypoxia-preconditioned gingiva-derived mesenchymal stem cell-conditioned medium accelerates burn wound healing
Source: Sci Rep. 2026 May 25;16:23486. doi: 10.1038/s41598-026-54462-8 (PMC13415783; doi:10.1038/s41598-026-54462-8)
Supplement: Supplementary file 3 — Supplementary Material 3 [file 41598_2026_54462_MOESM3_ESM.docx]

**Supplementary Table S2.** Key Resources and Reagents Used in the Study

| **Reagent / Material** | **Vendor (Location)** | **Catalog Number** | **Application in Study** |
| --- | --- | --- | --- |
| α-MEM medium | Gibco, Grand Island, NY, USA | 12571-063 | GMSC culture |
| DMEM medium | VivaCell, Shanghai, China | C3113-0500 | Osteogenic differentiation |
| RPMI-1640 medium | VivaCell, Shanghai, China | C3010-0500 | THP-1 cell culture |
| Fetal bovine serum (FBS) | Punosai, Shanghai, China | PAN100-500 | Cell culture supplementation |
| Penicillin–streptomycin | Beyotime, Shanghai, China | C0222 | Antibiotic supplementation |
| Trypsin (0.25%) | Biosharp | BL512A | Cell passaging |
| CD90 antibody | Proteintech, Wuhan, China | 66766-1-Ig ([PTGLab](https://www.ptglab.com/products/CD90-Antibody-66766-1-Ig.htm?srsltid=AfmBOoqofJ1cpeKC1l4dR6vwkd_lStHwZaKA0FYqGHI2qBXXum8xSBdy&utm_source=chatgpt.com" \o "CD90 antibody (66766-1-Ig))) | Immunofluorescence staining |
| CD105 antibody | Proteintech, Wuhan, China | 60158-1-Ig | Immunofluorescence staining |
| Fluorophore-conjugated secondary antibodies | Proteintech, Wuhan, China | SA00013-2 | Immunofluorescence staining |
| DAPI | Servicebio, Wuhan, China | G1012 | Nuclear staining |
| Triton X-100 | Beyotime, Shanghai, China | ST795 | Cell permeabilization |
| Goat serum | Servicebio, Wuhan, China | G1208 | Blocking reagent |
| Alizarin Red S | Solarbio, Beijing, China | G1452 | Osteogenic staining |
| Oil Red O | Servicebio, Wuhan, China | G1015 | Adipogenic staining |
| PMA | Sigma-Aldrich, USA | P8139 | THP-1 macrophage differentiation |
| IFN-γ | PeproTech, NJ, USA | 300-02 | M1 macrophage polarization |
| LPS | Sigma-Aldrich, USA | L2630 | M1 macrophage polarization |
| LY294002 | Selleck, USA | S1105 | PI3K pathway inhibition |
| TNF-α ELISA kit | 4ABio, Beijing, China | CHE0017 | Cytokine measurement |
| IL-1β ELISA kit | 4ABio, Beijing, China | CHE0001 | Cytokine measurement |
| TRIzol reagent | Invitrogen, Carlsbad, CA, USA | 15596026 | RNA extraction |
| Hifair® III First Strand cDNA Synthesis Kit | Yeasen, Shanghai, China | 11141ES60 | cDNA synthesis |
| SYBR Green Master Mix | Yeasen, Shanghai, China | 11201ES08 | RT-qPCR |
| RIPA lysis buffer | Servicebio, Wuhan, China | G2002 | Protein extraction |
| BCA Protein Assay Kit | Servicebio, Wuhan, China | G2026 | Protein quantification |
| SDS-PAGE gels | Epizyme Biotech, Shanghai, China | PG112 | Protein separation |
| PVDF membranes | Millipore, Burlington, MA, USA | IPVH00010 | Western blot transfer |
| p-AKT antibody | Abcam, Cambridge, UK | ab81283 | Western blot |
| AKT antibody | Proteintech, Wuhan, China | 10176-2-AP | Western blot |
| β-actin antibody | Proteintech, Wuhan, China | 66009-1-Ig | Western blot loading control |
| GAPDH antibody | Proteintech, Wuhan, China | 60004-1-Ig | Western blot loading control |
| HRP-conjugated secondary antibodies | Proteintech, Wuhan, China | SA00001-2 | Western blot |
| ECL substrate | Thermo Fisher Scientific, USA | 32106 | Signal detection |
| Pan-cytokeratin (Pan-CK) antibody | Abcam, Cambridge, UK | ab7753 | Immunohistochemistry |
| Tenascin-C (TNC) antibody | Abcam, Cambridge, UK | ab108930 | Immunohistochemistry |
| Hematoxylin and eosin staining kit | Servicebio, Wuhan, China | G1005 | Histological staining |
| Masson’s trichrome staining kit | Servicebio, Wuhan, China | G1006 | Collagen staining |
| DAB substrate kit | Servicebio, Wuhan, China | G1211 | IHC signal visualization |
| 0.22 μm filter membrane | Millipore, Burlington, MA, USA | SLGP033RS | Conditioned medium filtration |
| Transwell inserts (8 μm pore size) | Corning, NY, USA | 3422 | Cell migration assay |
| Crystal violet | Servicebio, Wuhan, China | G1063 | Transwell staining |
| Paraformaldehyde (4%) | Servicebio, Wuhan, China | G1101 | Cell/tissue fixation |
| StepOnePlus™ Real-Time PCR System | Applied Biosystems | 4376600 | RT-qPCR analysis |
| NanoDrop 2000 spectrophotometer | Thermo Fisher Scientific | ND-2000 | RNA quantification |
| Hypoxia chamber | Thermo Fisher Scientific, Waltham, MA, USA | 3131 | Hypoxic preconditioning |
| ImageJ software | NIH, Bethesda, MD, USA | Version 1.53 | Image quantification |
| GraphPad Prism software | GraphPad Software, San Diego, CA, USA | Version 9.0 | Statistical analysis |
